# Physiological, Proteomic, and Resin Yield-Related Genes Expression Analysis Provides Insights into the Mechanisms Regulating Resin Yield in Masson Pine

**DOI:** 10.3390/ijms241813813

**Published:** 2023-09-07

**Authors:** Zhengchun Li, Zijing Zhou, Qiandong Hou, Luonan Shen, Hong Zhao, Xiaopeng Wen

**Affiliations:** 1 Institute for Forest Resources & Environment of Guizhou, College of Forestry, Guizhou University, Guiyang 550025, China; 2 Key Laboratory of Plant Resource Conservation and Germplasm Innovation in Mountainous Region (Ministry of Education), Institute of Agro-Bioengineering, Guizhou University, Guiyang 550025, China; 3 Guizhou Key Lab of Agro-Bioengineering, Institute of Agro-Bioengineering, Guizhou University, Guiyang 550025, China

**Keywords:** masson pine, resin yield, proteomic, terpenoid biosynthesis, high resin-yielding cultivars

## Abstract

Masson pine (*Pinus massoniana* Lamb.) is an important resin-producing conifer species in China. Resin yield is a highly heritable trait and varies greatly among different genotypes. However, the mechanisms regulating the resin yield of masson pine remain largely unknown. In this study, physiological, proteomic, and gene expression analysis was performed on xylem tissues of masson pine with high and low resin yield. Physiological investigation showed that the activity of terpene synthase, as well as the contents of soluble sugar, jasmonic acid (JA), methyl jasmonate (MeJA), gibberellins (GA_1_, GA_4_, GA_9_, GA_19_, and GA_20_), indole-3-acetic acid (IAA), and abscisic acid (ABA) were significantly increased in the high yielder, whereas sucrose and salicylic acid (SA) were significantly decreased compared with the low one. A total of 2984 differentially expressed proteins (DEPs) were identified in four groups, which were mainly enriched in the biosynthesis of secondary metabolites, protein processing in the endoplasmic reticulum, carbohydrate metabolism, phytohormone biosynthesis, glutathione metabolism, and plant-pathogen interaction. Integrated physiological and proteomic analysis revealed that carbohydrate metabolism, terpenoid biosynthesis, resistance to stress, as well as JA and GA biosynthesis and signaling, play key roles in regulating resin yield. A series of proteins associated with resin yield, e.g., terpene synthase proteins (TPSs), ATP-binding cassette transporters (ABCs), glutathione S-transferase proteins (GSTs), and heat shock proteins (HSPs), were identified. Resin yield-related gene expression was also associated with resin yield. Our study unveils the implicated molecular mechanisms regulating resin yield and is of pivotal significance to breeding strategies of high resin-yielding masson pine cultivars.

## 1. Introduction

Masson pine (*Pinus massoniana* Lamb.) is one of the most important resin-producing conifer species in China, and approximately 90% of the resin is tapped from this species [1]. Resin, a complex mixture of different monoterpenes, sesquiterpenes, and diterpenes [2,3], is stored in resin ducts from stems, roots, needles, and reproductive structures [4,5]. As one of the most important non-wood forestry products, resin is widely used in industry, including chemicals, pharmaceuticals, agrochemicals, food additives, bioenergy, etc. [6,7]. Furthermore, resin also plays a key role in the conifer’s defense against insects and pathogens, including mechanical and chemical mechanisms that can be constitutively present or induced upon attack [3]. The constitutive resin flow in trees of pine species is a major feature of tree resistance to bark beetle attack [8]. Rapidly increasing resin yield in conifer stems via genomic selection and genetic engineering may enhance resistance to bark beetles and terpenoid yield for liquid biofuels [9]. Thereby, the enhancement of constitutive resin yield is an important goal for both genetic improvement and commercial plantation of conifers.

Resin yield is a quantitative trait under moderate to strong genetic control, and studies on maritime pine (*Pinus pinaster*), loblolly pine (*Pinus teada*), masson pine (*Pinus massoniana*), and slash pine (*Pinus elliottii*) have shown that genetic factors have a great influence on resin yield [10,11,12,13]. Previous studies have demonstrated that resin yield was associated with growth traits and morphologic traits, with the high resin-yielding trees tending to have a larger diameter and crown size compared with the low ones [12,14]. Resin yield was also related to resin flow rate, resin duct characteristics, resin components, sugar contents, and chlorophyll contents [15,16]. Significant genetic variations associated with resin yield have been found among different genotypes of masson pine, and extensive genetic gains can be achieved from the selection of high resin-yielding germplasm [12,17]. Elucidation of the genetic mechanisms of resin yield will provide a theoretical foundation for enhancing resin yield via breeding and cultivation measures. In conifers, terpenoid biosynthesis is rooted in two isoprenoid molecules, isopentenyl diphosphate (IPP) and dimethylallyl diphosphate (DMAPP). IPP and DMAPP are derived from the methylerythritol 4-phosphate (MEP) and mevalonate (MEV) pathways. DMAPP containing one, two or three IPP units are catalyzed using different isoprenyl diphosphate synthases (IDS) to produce the precursors’ geranyl diphosphate (GPP, C10), farnesyl diphosphate (FPP, C15), and geranylgeranyl diphosphate (GGPP, C20), respectively [18], then the corresponding monoterpenes, sesquiterpenes, and diterpenes are synthesized using terpene synthase (TPS) [3]. Cytochrome P450 monooxygenases (P450s) are involved in the further oxidation of resin diterpenes to form diterpene resin acids (DRAs); functionally characterized P450s of DRAs biosynthesis are members of the conifer-specific CYP720B subfamily [19,20,21]. TPS contributes the most to the structure diversity of resin terpenes, and P450s further increase the diversity of terpenes produced using TPS [22].

To date, transcriptomes have been widely used to explore the molecular regulation mechanism of resin yield, and a number of genes associated with resin yield have been screened [23,24,25,26,27]. However, transcript levels by themselves are not sufficient to predict protein levels in many cases since they do not take into account post-transcriptional processes and are, therefore, insufficient to explain the relationships between genotype and phenotype [28]. Proteins are thought to be more directly associated with metabolites than mRNAs [29]. Proteomics is an effective approach to investigating the function of proteins and their complex regulatory mechanisms [30]. Tandem mass tag (TMT), a quantitative protein technique with in vitro isotope labeling, is one of the most powerful proteomics technologies to identify and quantify proteins and has been extensively used in plant proteomics [31,32]. Nowadays, proteomics has been successfully applied to investigate the variations of protein components among different resin-yielding masson pines. Shi et al. [33] conducted a TMT-based proteomics analysis of secondary xylem tissues with high, medium, and low resin yield at the peak of resin production and identified candidate proteins involved in resin biosynthesis. Li et al. [16] carried out an iTRAQ-based proteomics analysis of needles with high and common resin yield at the peak of resin production and identified a series of candidate proteins and regulatory pathways associated with resin yield. Nevertheless, the limited information is still insufficient to explain the genetic mechanisms of resin yield. The dynamic changes of protein expression of masson pine with high and low resin yield at different resin production stages have not been reported.

To better understand the mechanisms regulating resin yield, a physiological investigation was carried out between high and low resin-yielding masson pines; subsequently, TMT-based proteomics was employed to analyze protein expression profiles of masson pine with high and low resin yield at two resin production stages. Functional annotation, KEGG pathway enrichment, and hierarchical clustering analysis were performed to identify candidate proteins and regulatory pathways associated with resin yield. Finally, relative gene expression analysis of resin production-related genes among various resin-yielding masson pines was performed using quantitative real-time PCR. This study further betters the understanding of mechanisms regulating resin yield and provides valuable genetic resources for breeding high resin-yielding masson pine cultivars.

## 2. Results

### 2.1. Physiological Characteristics of High and Low-Resin-Yielding Masson Pines

**Figure 1:**
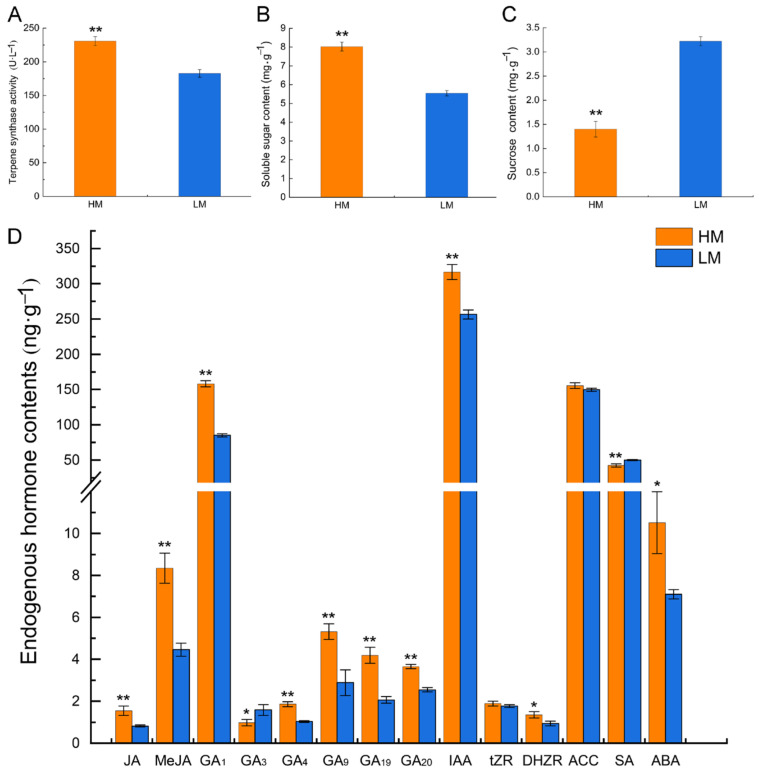
Comparison of physiological characteristics in terpene synthase activity (**A**), soluble sugar content (**B**), sucrose content (**C**), and endogenous hormone contents (**D**) between the high and low resin-yielding masson pines. The xylem tissues (fresh weight) were used for the experiment. “HM” means the high resin yielder, and “LM” means the low resin yielder. Data were shown as mean ± standard deviation from three biological replicates. * and ** indicate significant differences between the high and low ones at *p* < 0.05 and *p* < 0.01, respectively. JA: jasmonic acid; MeJA: methyl jasmonate; GA_1_: gibberellin A_1_; GA_3_: gibberellin A_3_; GA_4_: gibberellin A_4_; GA_9_: gibberellin A_9_; GA_19_: gibberellin A_19_; GA_20_: gibberellin A_20_; IAA: indole-3-acetic acid; tZR: trans-zeatin riboside; DHZR: dihydrozeatin ribonucleoside; ACC: 1-aminocyclopropanecarboxylic acid; SA: salicylic acid; ABA: abscisic acid.

To unravel the physiological characteristics of high and low resin-yielding masson pines, terpene synthase (TPS) activity, soluble sugar, and sucrose contents, as well as the contents of jasmonates (JAs), gibberellins (GAs), auxin, cytokinins (CTKs), ethylene biosynthesis precursor 1-aminocyclopropanecarboxylic acid (ACC), salicylic acid (SA), and abscisic acid (ABA) were quantified between the high and low resin yielders. The TPS, soluble sugar, and sucrose showed significant differences between them (Figure 1A–C). Terpene synthases are important enzymes involved in terpenoid biosynthesis, and their activity could reflect the level of terpenoid biosynthesis. The activity of TPS increased by 26% in the high resin yielder compared with the low one. The content of soluble sugar increased by 45%, whereas the content of sucrose decreased by 57% in the high resin yielder compared with the low one.

In total, 14 phytohormones were detected (content > 1 ng·g^−1^), including JAs (jasmonic acid, JA and methyl jasmonate, MeJA), GAs (GA_1_, GA_3_, GA_4_, GA_9_, GA_19_, and GA_20_), auxin (indole-3-acetic acid, IAA), CTKs (trans-zeatin riboside, tZR, and dihydrozeatin ribonucleoside, DHZR), ACC, SA, and ABA. JAs, GAs, IAA, DHZR, SA, and ABA showed significant differences between the high and low resin yielders, while DHZR and ACC showed no significant differences between them (Figure 1D). Compared with the low resin yielder, JA, MeJA, GA_1_, GA_4_, GA_9_, GA_19_, GA_20_, IAA, DHZR, and ABA accumulated more, whereas GA_3_ and SA were decreased in the high one. The contents of JA, MeJA, GA_1_, GA_4_, GA_9_, GA_19_, GA_20_, IAA, DHZR, and ABA in the high resin yielder were 1.89, 1.87, 1.86, 1.79, 1.85, 2.03, 1.44, 1.23, 1.44, and 1.48 times higher than those in the low one, respectively. Conversely, the contents of GA_3_ and SA in the high resin yielder were 0.62 and 0.85 times those in the low one, respectively.

### 2.2. Proteome Profiling and DEPs Identification

**Figure 2:**
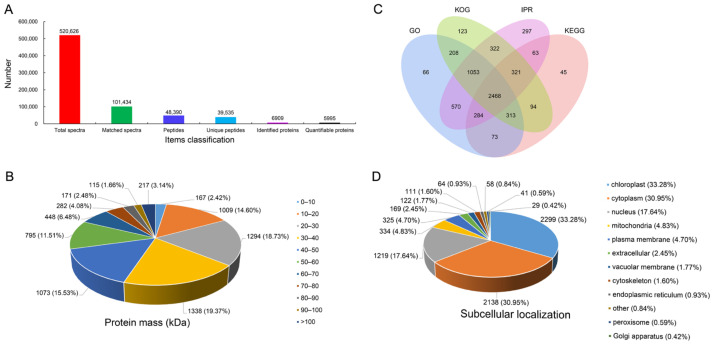
Identification and analysis of the proteins in masson pine based on proteome profiles. (**A**) Number of spectra, peptides, and proteins. (**B**) Distribution of molecular mass of the identified proteins. (**C**) Proteins functional annotation in four databases (GO, KEGG, KOG, and IPR). (**D**) Subcellular localization of the identified proteins.

A total of 520,626 spectra were obtained using mass spectrometry, of which 101,434 available spectra were matched using LC-MS/MS spectra database search analysis, with a utilization rate of 19.5% of the spectra. In total, 48,390 peptides, 39,535 unique peptides, and 6909 proteins were identified, respectively. After filtering the data, 5995 proteins were confidently identified and quantified (Figure 2A). The molecular mass of the identified proteins showed that about 80% of the proteins had masses between 10 and 60 kDa (Figure 2B). Furthermore, GO, KEGG, KOG, and IPR databases were used for functional annotation of the identified proteins. A total of 6300 proteins were annotated, of which 5035, 3661, 4902, and 5378 proteins were annotated to GO, KEGG, KOG, and IPR databases, respectively, and 2468 proteins were annotated simultaneously in four databases (Figure 2C). Additionally, subcellular localization showed that most of the identified proteins were localized in the chloroplasts (2299, 33.28%), cytoplasm (2138, 30.95%), nucleus (1219, 17.64%), and mitochondria (334, 4.83%) (Figure 2D).

**Figure 3:**
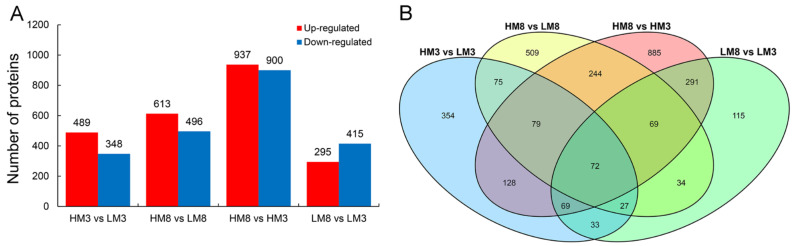
DEPs in four comparison groups of masson pine. (**A**) Number of up- and down-regulated DEPs in four comparison groups. (**B**) Venn diagram showing the overlap of DEPs among four comparison groups. “HM” and “LM” represent the high and low resin yielders, respectively. Samples denoted by the number “3” are collected in March, representing the lowest level of the species resin production. Samples collected in August are denoted by the number “8”, representing the highest level of the species resin production. Among the four comparison groups, LM3, LM8, HM3, and LM3 are the controls of HM3 vs. LM3, HM8 vs. LM8, HM8 vs. HM3, and LM8 vs. LM3, respectively.

In total, 2984 proteins were identified among the four paired comparisons (Table S1). A relatively higher number of DEPs was observed in HM8 vs. LM8 (1109) compared with HM3 vs. LM3 (837), and the number of DEPs in HM8 vs. HM3 (1837) was greatly higher than that in LM8 vs. LM3 (710) (Figure 3A). Of these, 253 were common in both HM3 vs. LM3 and HM8 vs. LM8, 501 were common in both HM8 vs. HM3 and LM8 vs. LM3, and 72 were common in all comparison groups, indicating that these proteins may influence the resin yield. A total of 354, 509, 885, and 115 were specifically differentially expressed in HM3 vs. LM3, HM8 vs. LM8, HM3 vs. LM3, and LM8 vs. LM3, respectively (Figure 3B). Further, volcano plots were used to show protein expression changes in each comparison group, with most up-regulated DEPs being enriched in HM8 vs. LM8 and HM8 vs. HM3 (Figure S1).

### 2.3. Function Classification and Metabolic Pathways Enrichment Analysis of DEPs

In order to reveal the main biological processes regulating resin yield, GO enrichment was used for the functional classification of DEPs in each comparison group, including biological process, cellular component, and molecular function (Figure S2). GO enrichment analysis demonstrated that the main differences among the four groups were focused on biological process and molecular function. In the biological processes, most DEPs were enriched in the metabolic process, cellular process, single-organism process, localization, response to stimulus, and biological regulation. Within the molecular functions, DEPs were mainly distributed in catalytic activity and binding.

**Figure 4:**
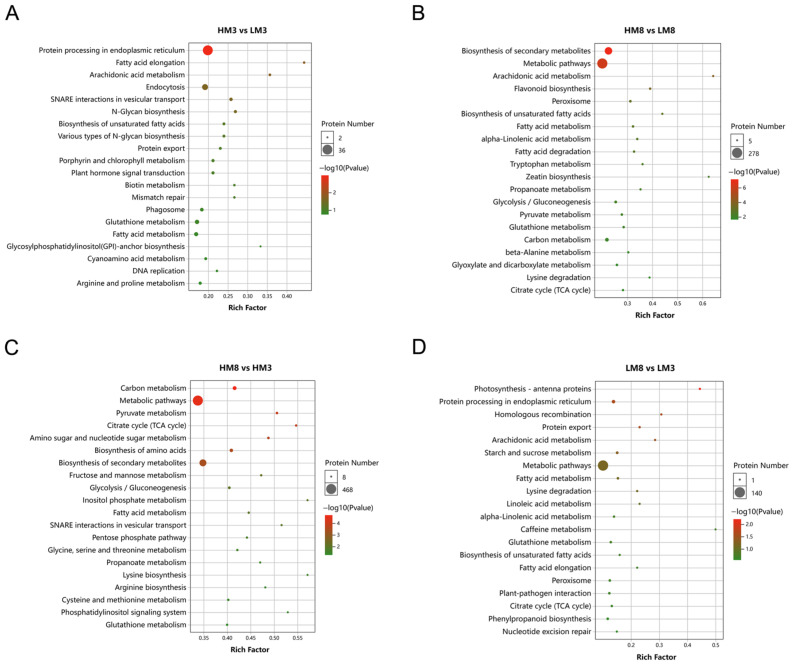
KEGG enrichment pathways of DEPs in HM3 vs. LM3 (**A**), HM8 vs. LM8 (**B**), HM8 vs. HM3 (**C**), and LM8 vs. LM3 (**D**).

To further analyze the key metabolic pathways associated with resin yield, KEGG enrichment analysis was performed on DEPs in each group (Figure 4). The results showed that DEPs were involved in multiple metabolic pathways, many of which were enriched in the biosynthesis of secondary metabolites among four comparison groups. In addition, we found that protein processing in the endoplasmic reticulum pathway was the most significantly enriched in HM3 vs. LM3. In HM8 vs. LM8, most DEPs were significantly enriched in the biosynthesis of secondary metabolites, metabolic pathways, arachidonic acid metabolism, flavonoid biosynthesis, peroxisome, biosynthesis of unsaturated fatty acids, fatty acid metabolism, alpha-linolenic acid metabolism, fatty acid degradation, tryptophan metabolism, and zeatin biosynthesis. In HM8 vs. HM3, DEPs were mainly distributed in carbon metabolism, metabolic pathways, pyruvate metabolism, citrate cycle, amino sugar, and nucleotide sugar metabolism, biosynthesis of amino acids, and biosynthesis of secondary metabolites. Protein processing in the endoplasmic reticulum was the significantly enriched pathway containing the most DEPs in LM8 vs. LM3.

### 2.4. DEPs Expression Trend Analysis using Mfuzz Clustering

**Figure 5:**
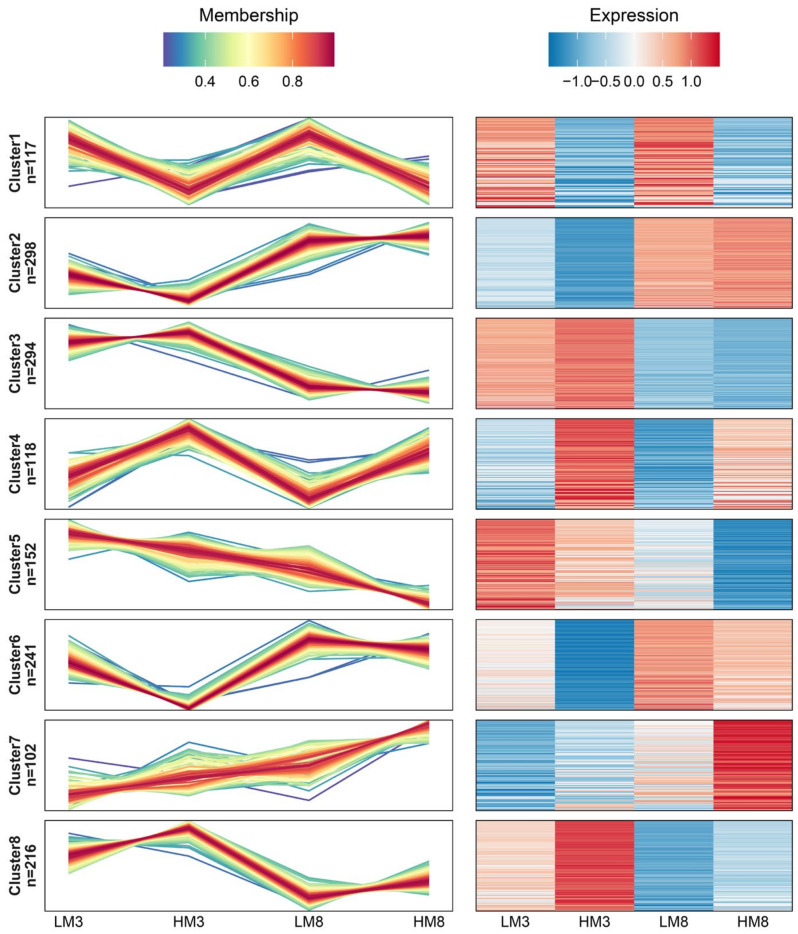
Mfuzz clustering diagram of DEPs in samples LM3, HM3, LM8, and HM8. The left side of the graph represents the line chart of protein expression, and the right side represents the heatmap of protein expression. Line chart: The horizontal axis is the sample, the ordinate is the relative expression of the protein, one broken line represents one protein, and the line color indicates the membership of the protein in the current class. Heatmap: The horizontal coordinate is the sample, the ordinate is a different protein, and the color of the heatmap represents the relative expression of the protein in the sample.

Hierarchical clustering analysis was performed on the DEPs expression profiles using Mfuzz clustering to investigate how they responded to the resin yield. In total, 1538 DEPs were grouped into eight distinct clusters, and the proteins with similar expression patterns tended to have similar functions (Figure 5, Table S2). Cluster 4 and cluster 1 represent most proteins that were up- and down-regulated in samples HM3 and HM8 compared with LM3 and LM8, respectively. Clusters 2 and 6 represent most proteins that were up-regulated in samples HM8 and LM8 compared with HM3 and LM3, and clusters 3 and 8 represent most proteins that were down-regulated in samples HM8 and LM8 compared with HM3 and LM3. Cluster 7 and cluster 5 represent most proteins that were up- and down-regulated in sample HM8 compared with LM8, HM3, and LM3, respectively. Among these, clusters 4 and 7 showed a similar tendency with resin yield, and clusters 1 and 5 showed the opposite tendency with resin yield. These indicated that clusters 4 and 7 may positively correlate with resin yield, while clusters 1 and 5 may negatively correlate with resin yield. To further analyze the potential functions of these DEPs, the KEGG enrichment analysis was carried out. The results showed that most DEPs were enriched in the biosynthesis of secondary metabolites, glycolysis/gluconeogenesis, citrate cycle, protein processing in the endoplasmic reticulum, glutathione metabolism, and plant-pathogen interaction, indicating that these pathways may be involved in regulating resin yield (Figure S3).

To determine the proteins related to resin yield in the above four clusters, we further analyzed the protein expression profiles in the four comparison groups and identified a set of proteins closely correlated with resin yield (Table S3). In cluster 4, 23 proteins were up-regulated in both HM3 vs. LM3 and HM8 vs. LM8, e.g., glutathione S-transferase (GST) and U6 snRNA-associated Sm-like protein (LSm6). In cluster 7, 15 proteins were simultaneously up-regulated in the four comparisons HM3 vs. LM3, HM8 vs. LM8, HM8 vs. HM3, and LM8 vs. LM3, e.g., UDP-glycosyltransferase 91A1 (UGT91A1) and calcium-binding protein (CML). In cluster 1, 25 proteins were down-regulated in both HM3 vs. LM3 and HM8 vs. LM8, e.g., small heat shock protein (HSP22) and 25.3 kDa heat shock protein (HSP25.3). In cluster 5, 7 proteins were simultaneously down-regulated in these four comparison groups, e.g., 20 kDa chaperonin (CPN20) and lipase-like (PAD4).

### 2.5. Identification of DEPS Involved in Resin Yield

**Figure 6:**
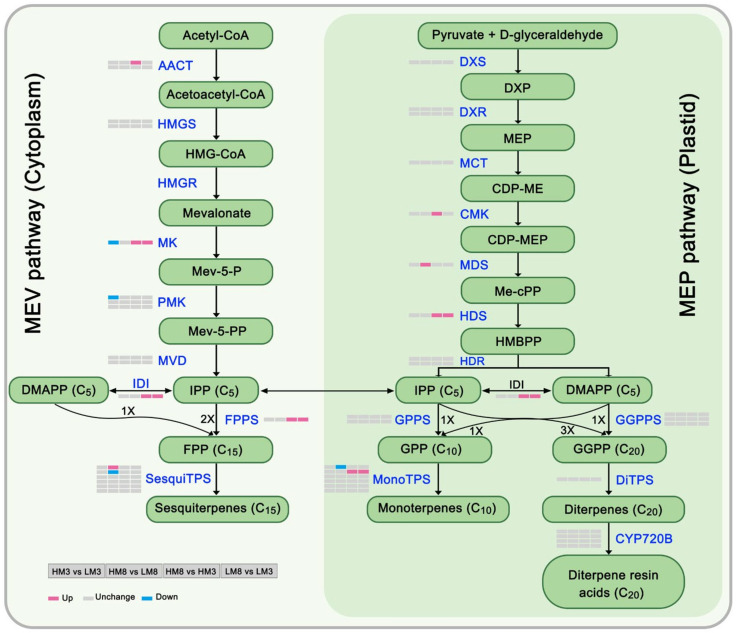
Heatmap of DEPs participated in the mevalonate (MEV) and methylerythritol phosphate (MEP) pathways of terpenoid biosynthesis. Up- and down-regulated DEPs were indicated by red and blue colors, respectively. Nonsignificant proteins were indicated by a gray color. The cells (from left to right) in the heatmap represent the comparison groups of HM3 vs. LM3, HM8 vs. LM8, HM8 vs. HM3, and LM8 vs. LM3, respectively. AACT: acetyl-CoA C-acetyltransferase; HMGS: hydroxymethylglutaryl-CoA synthase; HMGR: hydroxymethylglutaryl-CoA reductase; MK: mevalonate kinase; PMK: phosphomevalonate kinase; MVD: diphosphomevalonate decarboxylase; DXS: 1-deoxy-D-xylulose-5-phosphate synthase; DXR: 1-deoxy-D-xylulose-5-phosphate reductoisomerase; MCT: 2-C-methyl-D-erythritol 4-phosphate cytidylyltransferase; CMK: 4-diphosphocytidyl-2-C-methyl-D-erythritol kinase; MDS: 2-C-methyl-D-erythritol 2, 4-cyclodiphosphate synthase; HDS: 4-hydroxy-3-methylbut-2-enyl-diphosphate synthase; HDR: 4-hydroxy-3-methylbut-2-enyl-diphosphate reductase; IDI: isopentenyl-diphosphate Delta-isomerase; FPPS: farnesyl pyrophosphate synthase; GPPS: geranyl diphosphate synthase; GGPPS: geranylgeranyl diphosphate synthase; TPS: terpene synthase; CYP720B: Cytochrome 720B.

Among all identified proteins, 43 proteins were annotated to be associated with terpenoid biosynthesis, including 26 terpenoid backbone biosynthesis-related proteins, 13 terpene synthases, and 4 CYP20Bs (Figure 6, Table S4). A total of 12 DEPs were identified among four comparison groups. It was found that mevalonate kinase (MK), 4-hydroxy-3-methylbut-2-enyl-diphosphate synthase (HDS), isopentenyl-diphosphate Delta-isomerase (IDI), farnesyl diphosphate synthase (FPPS), and α-pinene synthase (MonoTPS2) were up-regulated in both HM8 vs. HM3 and LM8 vs. LM3. In addition, MK and phosphomevalonate kinase (PMK) were down-regulated in HM3 vs. LM3. In HM8 vs. LM8, 2-C-methyl-D-erythritol 2,4-cyclodiphosphate synthase (MDS) and 5-germacradien-4-ol synthase (SesquiTPS1) were greatly up-regulated, whereas longifolene synthase (SesquiTPS2) and β-pinene synthase (MonoTPS1) were greatly down-regulated. Acetyl-CoA C-acetyltransferase (AACT) and 4-diphosphocytidyl-2-C-methyl-D-erythritol kinase (CMK) were also up-regulated in HM8 vs. HM3. In LM8 vs. LM3, all DEPs were simultaneously up-regulated, including MK, HDS, FPPS, IDI, and MonoTPS2.

**Figure 7:**
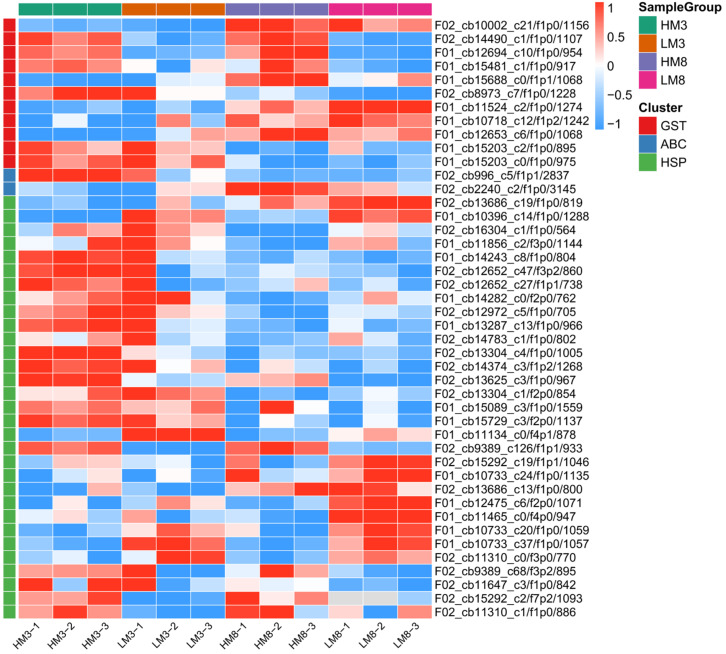
Heatmap of DEPs referred to GSTs, ABCs, and HSPs in different samples (excluding a few proteins that were not expressed in all samples).

In total, there were 14, 5, and 37 DEPs participated in glutathione metabolism, ABC transporters, and protein processing in the endoplasmic reticulum, including 14 glutathione S-transferases (GSTs), 5 ATP-binding cassette transporters (ABCs), and 37 heat shock proteins (HSPs) among four groups (Figure 7, Table S5). Among all GSTs, there were 5, 7, 8, and 4 DEPs in HM3 vs. LM3, HM8 vs. LM8, HM3 vs. LM3, and LM8 vs. LM3, respectively. Most GSTs were up-regulated in the four comparison groups, and two proteins were up-regulated in both HM3 vs. LM3, and HM8 vs. LM8. Among all ABCs, there were 2, 3, and 1 DEPs in HM3 vs. LM3, HM8 vs. LM8, and HM8 vs. HM3, respectively, and four proteins were up-regulated in HM3 vs. LM3 and HM8 vs. LM8. Among all HSPs, there were 10, 17, 15, and 12 DEPs in HM3 vs. LM3, HM8 vs. LM8, HM8 vs. HM3, and LM8 vs. LM3, respectively. The two and four HSPs were up- and down-regulated in both HM3 vs. LM3 and HM8 vs. LM8, respectively. Most of the DEPs referred to HSPs exhibited high fold changes, including six up-regulated and six down-regulated proteins, all of which were more than 4-fold.

**Figure 8:**
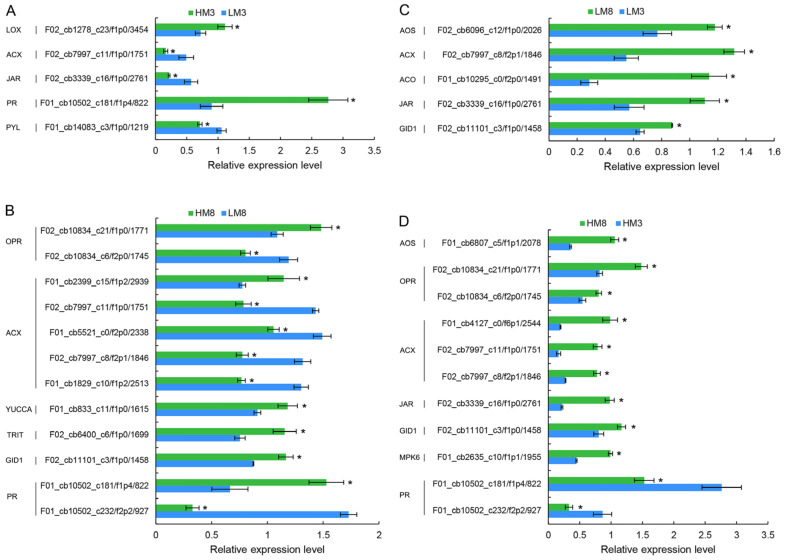
Relative expression level of DEPs involved in plant hormone biosynthesis and signal transduction in HM3 vs. LM3 (**A**), HM8 vs. LM8 (**B**), LM8 vs. LM3 (**C**), and HM8 vs. HM3 (**D**). * indicates a significant difference at *p*-value < 0.05. LOX: lipoxygenase; ACX: acyl-coenzyme A oxidase; JAR: jasmonic acid-amino synthetase; PR: pathogenesis-related protein; PYL: abscisic acid receptor PYL; OPR: 12-oxophytodienoic acid reductase; YUCCA: indole-3-pyruvate monooxygenase; TRIT: tRNA dimethylallyltransferase; GID1: gibberellin receptor GID1-like protein; AOS: allene oxide synthase; ACO: aminocyclopropanecarboxylate oxidase; MPK6, mitogen-activated protein kinase 6.

In total, 120 DEPs were annotated to be involved in plant hormone biosynthesis and signal transduction pathways among four comparison groups, of which 33, 61, 79, and 25 were differentially expressed in HM3 vs. LM3, HM8 vs. LM8, HM8 vs. HM3, and LM8 vs. LM3, respectively (Figure 8, Table S6). In HM3 vs. LM3, lipoxygenase (LOX) involved in JA biosynthesis was significantly up-regulated, whereas acyl-coenzyme A oxidase (ACX) involved in JA biosynthesis was significantly down-regulated. Furthermore, pathogenesis-related protein (PR) involved in SA signaling was significantly up-regulated. Conversely, jasmonic acid-amino synthetase (JAR) involved in JA signaling and abscisic acid receptor PYL involved in ABA signaling were significantly down-regulated. In HM8 vs. LM8, one 12-oxophytodienoic acid reductase (OPR) and one ACX related to JA biosynthesis, together with tRNA dimethylallyltransferase (TRIT) related to CTK biosynthesis and indole-3-pyruvate monooxygenase (YUCCA) related to auxin biosynthesis were greatly up-regulated. In addition, gibberellin receptor GID1-like protein (GID1) related to GA signaling and one PR were dramatically up-regulated. In HM8 vs. HM3, allene oxide synthase (AOS), two OPRs, and three ACXs associated with JA biosynthesis all greatly up-regulated. Further, JAR and GID1 together with mitogen-activated protein kinase 6 (MPK6) associated with ethylene signaling, were greatly up-regulated, while two PRs associated with SA signaling were greatly down-regulated. In LM8 vs. LM3, AOS, ACX, and JAR participated in JA biosynthesis and signaling, together with aminocyclopropanecarboxylate oxidase (ACO) participated in ethylene biosynthesis, and GID1 participated in GA signaling were obviously up-regulated.

### 2.6. Change Patterns of Resin Yield-Related Genes between High and Low Resin Resin-Yielding Masson Pines

**Figure 9:**
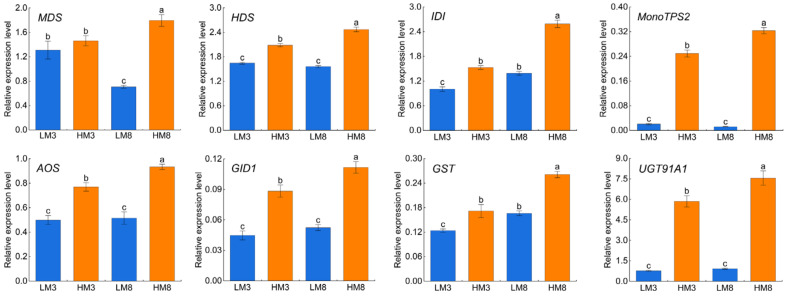
Expression analyses by qRT-PCR for resin yield-related genes in masson pine with high and low resin yield. Bars indicate the mean ± standard error (*n* = 3). Different lowercase letters in the figure indicate significant differences at *p* < 0.05. *MDS*: 2-C-methyl-D-erythritol 2,4-cyclodiphosphate synthase; *HDS*: 4-hydroxy-3-methylbut-2-enyl-diphosphate synthase; *IDI*: isopentenyl-diphosphate Delta-isomerase; *MonoTPS2*: α-pinene synthase; *AOS*: allene oxide synthase; *GID1*: gibberellin receptor GID1-like protein; *GST*: glutathione S-transferases; *UGT91A1*: UDP-glycosyltransferase 91A1.

To illustrate the changes in the expression of resin yield-related genes between the high and low resin yielders, quantitative real-time PCR (qRT-PCR) analysis was performed for the selected eight genes (Figure 9). Among these genes, *MDS*, *HDS*, and *IDI* are involved in terpenoid backbone biosynthesis, *MonoTPS2* is involved in resin monoterpene biosynthesis, as well as *AOS*, *GID1*, *GST*, and *UGT91A1* may be involved in signaling regulation of resin biosynthesis. As shown in Figure 9, eight resin yield-related genes were significantly up-regulated in the high resin yielder compared with the low one. Among these genes, except for *MDS*, the other seven genes were up-regulated in both HM3 vs. LM3 and HM8 vs. LM8, implying the up-regulation of these genes might result in the increase in resin yield. Notably, *MonoTPS2* and *UGT91A1* showed dramatically increased abundance in the high resin yielder compared with the low one, strongly indicating their involvement in regulating resin yield. By further analyzing the expression relationship between these eight genes and their corresponding proteins, we found that change patterns of resin yield-related genes showed a similar trend with their corresponding proteins between the high and low resin yielders, suggesting that these genes were involved in regulating resin yield at the transcriptional level.

### 2.7. Parallel Reaction Monitoring Validation

**Figure 10:**
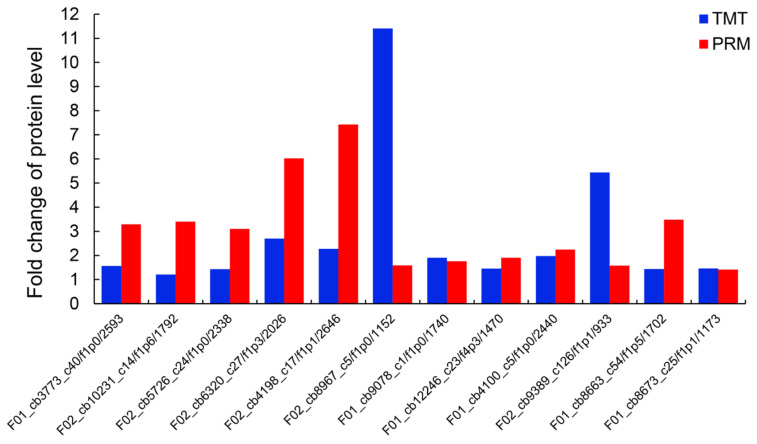
Validation of 12 selected DEPs in TMT results using parallel reaction monitoring (PRM). The ordinate represents the HM/LM ratio value, ratio > 1 represents the up-regulation, and ratio < 1 represents the down-regulation.

To validate the reliability of DEPs analysis in TMT results, parallel reaction monitoring (PRM) was performed on key enzymes that showed significant differences between the high and low resin yielders (Figure 10, Table S7). A total of 12 proteins, mainly involved in glycolysis/gluconeogenesis, starch and sucrose metabolism, citrate cycle, flavonoid biosynthesis, protein processing in endoplasmic reticulum, and glutathione metabolism, were selected for analysis. The results of PRM showed that the expression levels of all candidate proteins were consistent with the TMT results, indicating that proteome data are reliable.

## 3. Discussion

### 3.1. Carbohydrate Metabolism and Terpenoid Biosynthesis Regulate Resin Yield via Promoting Resin Biosynthesis

Higher plants produce triose phosphates using photosynthetic carbon fixation, and then triose phosphates are converted to sucrose, an important product of photosynthesis. Sucrose can be further broken down into glucose and fructose, which undergo a series of transformations to produce pyruvate, glycer-aldehyde-3 phosphate, and acetyl-CoA and further synthesize terpenoids and other secondary substances [34,35]. Previous studies have shown that the yield of terpenoids was tightly corrected with the content of some sugars in woody plants. The correlation between resin yield and soluble sugar content of needles reached a highly significant level in masson pine [36]. In rubber trees, carbohydrate metabolism provides abundant energy and substrates for latex regeneration, and its efficiency is a critical factor in rubber yield. Sucrose is a precursor of rubber, the loading and degradation ratio of which directly affects rubber productivity [37,38]. The sucrose content in laticifers of high-yielding rubber trees is lower than that of low-yielding rubber trees [39]. Our results showed that soluble sugar accumulated more, while sucrose was significantly decreased in the high resin-yielding masson pines compared with the low ones, suggesting that sugars, especially sucrose, may influence resin yield by regulating the substrates for terpenoid biosynthesis.

Terpene synthases directly affect terpenoid biosynthesis [40]. TPS activity in the high resin yielder was greatly higher than that in the low one, indicating that TPS may play an important role in regulating resin yield. Several genes encoding TPS have been reported to be closely correlated with resin yield in masson pine. The gene encoding α-pinene synthase was up-regulated in the high resin yielder, while tricyclene synthase showed a lower transcript expression level compared with the low one [23]. Terpenoid backbone biosynthesis-related proteins have also been reported to be involved in resin yield. Mei et al. [1] revealed that transcript levels of HMGR, MDS, HDS, and IDI showed higher expression in the high resin yielder, which could be used as the critical targets for the molecular-assisted selection of high resin-yielding germplasm in masson pine. In the present study, MDS and SesquiTPS1 were up-regulated, whereas MK and PMK were down-regulated in the high resin yielder, which was consistent with expression trends in needles of high resin-yielding masson pine [16]. Therefore, MDS and SesquiTPS1 may be positively involved in regulating resin yield. In addition, we found that all DEPs were up-regulated in HM8 vs. HM3 and LM8 vs. LM3, including AACT, CMK, HDS, IDI, FPPS, and MonoTPS2. Notably, HDS, IDI, FPPS, and MonoTPS2 were simultaneously up-regulated in HM8 vs. HM3 and LM8 vs. LM3, suggesting that these proteins were highly correlated with resin yield.

### 3.2. ABC and GST Improve Resin Yield via Facilitating Resin Transport

In conifers, the resin is transported from living cells in the stem to resin ducts, and when the stem is subjected to abiotic stimuli, the resin flows from the injured site [41]; however, the mechanisms of resin transport remain unclear. The ATP-binding cassette transporters (ABCs) are proteins that participate in special metabolite transport, such as flavonoids, anthocyanins, and terpenoids. *NpABC1* was the first reported transporter involved in terpenoid secretion from *Nicotiana plumbaginfolia* cells [42]. It has been proved that ABC transporters were involved in the transport of resin terpenes in *Pinus taeda* and *Pinus elliottii* [9,43]. Furthermore, previous studies have revealed that ABCs were associated with resin yield in masson pine [23,25]. In this study, the results of protein expression profiles also confirmed that the expression of several ABCs was significantly associated with resin yield, which was consistent with proteomic analysis of the high, medium, and low resin-yielding capacity of masson pine [33], indicating the important role of ABC transporters in regulating resin yield.

The glutathione S-transferases are ubiquitous and multifunctional binding proteins that are thought to play multiple functional roles in plants, such as in glucosinolate biosynthesis and metabolism [44], herbicide detoxification, plant developmental processes, signal transduction, and resistance to stress [45,46,47]. Importantly, in recent years, GSTs have been proven to play an important role in anthocyanin transport and accumulation, including in anthocyanin sequestration from the cytoplasm to the vacuole [48,49]. Here, we found that the expression of many GSTs was highly correlated with resin yield, with most GSTs exhibiting a higher expression in the high resin yielder compared with the low one. These indicated that GSTs may improve resin yield via mediating resin terpenes transport.

### 3.3. Stress-Related Proteins Involve in Resin Yield via Regulating Responses to Stress

Heat shock proteins (HSPs) are stress-related proteins that play important roles in multiple stresses, such as heat, drought, salt, cold, high light, and oxidative stress [50]. Several HSPs were previously proved to be involved in the conifer defense. Lippert et al. [51] revealed that 7 HSPs were induced to be overexpressed at the protein level by weevil feeding in Sitka spruce. Verne et al. [52] demonstrated that 15 HSPs were constitutively down-regulated in the resistant trees compared with the susceptible trees in spruce. Our study showed that 37 of 66 putative HSPs were differentially expressed in all comparison groups and that 10 of 17 differential HSPs were down-regulated in HM8 vs. LM8. Notably, 13 of 15 differential DEPs were down-regulated in HM8 vs. HM3. Thus, our results showed that the expression of HSPs was highly correlated with resin yield, suggesting that HSPs may affect the resin yield by regulating resistance to stress. The underlying mechanism is yet to be discovered. In addition, several proteins involved in plant-pathogen interaction were also found to be associated with resin yield, including PR protein and calcium-binding protein (CML). PR proteins are involved in antifungal activity and defense response in plants [53]. CML may influence stress response by mediating signal transduction and Ca homeostasis [54]. In this study, many stress-related proteins were closely correlated with resin yield, possibly because high resin yielders have other characteristics associated with an overall strong defense phenotype.

### 3.4. Plant Hormones Highly Regulate Resin Yield via Modulating Terpenoid Biosynthesis as Well as Xylem Growth and Differentiation

JA is an important phytohormone, which is widely involved in growth and development, metabolic regulation, and stress and defense responses in plants [55,56]. In the present study, we found that several key proteins involved in JA biosynthesis were significantly associated with resin yield. LOX, OPR, and ACX were up-regulated in the high resin yielder, which was consistent with increasing JAs levels in the high one. Notably, all DEPs involved in JA biosynthesis and signaling were up-regulated in both HM8 vs. HM3 and LM8 vs. LM3, including AOS, OPR, ACX, and JAR, strongly indicating that JA may also play a key role in constitutively regulating resin biosynthesis. Conversely, SA accumulated less in the high resin yielder compared with the low one. A previous study suggested that there may be some antagonism between JA and SA-mediated signaling in spruce [57]. Our hormone profiling data showed that SA was decreased with the increase in JAs levels, suggesting that JA and SA may be some antagonism in regulating resin yield. GA is a diterpenoid phytohormone that promotes secondary growth and xylem differentiation in many plants, e.g., *Arabidopsis*, potato, poplar, and hybrid aspen [58,59]. Previous studies have also shown that GA induced the expression of monoterpene synthase in *Salvia officinalis*, and an increase and decrease in the concentration of essential oil were found with increasing GA levels and blocked GA biosynthesis, respectively [60]. In this study, GA levels were positively associated with resin yield, suggesting that GA may regulate resin yield by modulating xylem differentiation as well as terpenoid biosynthesis. GID1, a GA receptor protein involved in GA signaling [61], was co-overexpressed in HM8 vs. LM8, HM8 vs. HM3, and LM8 vs. LM3, strongly indicating that GID1 may be a key protein involved in regulating resin yield. Auxin may be participated in stem structure formation, wood radial growth, and resin ducts in conifers [43]. It has been reported that exogenous application of auxin stimulant paste promoted resin production in slash pine [62]. In the current study, IAA level was significantly elevated in the high resin yielder, implying that auxin may regulate resin yield by affecting xylem growth and resin duct development. YUCCA, a key protein involved in auxin biosynthesis [63], was significantly up-regulated in the high resin yielder, which was consistent with the increasing IAA level, indicating that YUCCA may be involved in regulating resin yield. ABA is an important terpenoid phytohormone involved in many developmental processes and responses to environmental stresses and pathogens in plants [64]. Here, an increasing ABA level was found in the high resin yielder, suggesting that ABA may influence resin yield.

## 4. Materials and Methods

### 4.1. Plant Materials and Growth Conditions

The samples were acquired from the State Key Base of Improved Forest Varieties of Masson Pine (23°10′ N, 108°00′ E), Wuming County, Guangxi, China, which belongs to the subtropical monsoon climate with sufficient light, heat, and rainfall. The annual average temperature is 21.7 °C, the hottest can reach 40.7 °C, the coldest is as low as −0.8 °C, and the annual average rainfall is 1100–1700 mm. The resin yield was determined using the bark streak method of wounding for resin tapping [12] and calculated as the yield of the individual per day per 10 cm cutting surface width in grams. Based on the yield, two twelve-year-old variants, i.e., high resin yield (more than 15.0 g·d^−1^·10 cm^−1^) and low resin yield (less than 5.0 g·d^−1^·10 cm^−1^), were obtained from the same environment conditions and management. Three clonal ramets for each variant were used as three biological replicates. The bark was removed before sample collection, and the xylem tissues were harvested from the trunk one meter above the ground with a chisel into the xylem, 5 cm in length, 2 cm in width, and 2 mm in thickness. The xylem tissues of high and low resin-yielding variants were collected in March (the lowest level of the species resin production) and August (the highest level of the species resin production). The samples were collected at 11:00–12:00 a.m., immediately frozen in liquid nitrogen, and then stored at −80 °C refrigerator for physiological, proteomic, and gene expression analysis. “HM” represents samples collected from the high resin-yielding variant, and “LM” represents samples collected from the low resin-yielding variant. Samples collected in March are denoted by the number “3”, and samples collected in August are denoted by the number “8”.

### 4.2. Determination of Terpene Synthase Activity

The six independent xylem tissues collected from high- and low-resin-yielding variants in August were used for the measurement of terpene synthase (TPS) activity. The sample was ground into powder under liquid nitrogen, and then 500 mg of powder was accurately weighed and extracted with 4.5 mL of 0.01 mol·L^−1^ phosphate-buffered saline (PBS, pH = 7.4) (Meilun Biotechnology Co., Ltd., Dalian, China). The mixed solution was quickly ground to a complete homogenate. After centrifugation at 5000× *g* for 10 min at 4 °C, the supernatant was carefully collected for TPS activity analysis. TPS activity was determined using the plant TPS ELISA kit (Lanpai Biotechnology Co., Ltd., Shanghai, China) according to the manufacturer’s instructions. The measurement was performed on a Multiskan FC microplate reader (Thermo Scientific, Waltham, MA, USA), and each measurement was repeated three times.

### 4.3. Measurement of Soluble Sugar and Sucrose Contents

The six independent xylem tissues collected from high- and low-resin-yielding variants in August were used for the measurement of soluble sugar and sucrose contents. The soluble sugar content kit and the sucrose content kit (micro method) (Comin Biotechnology Co., Ltd., Suzhou, China) were used to determine soluble sugar and sucrose, respectively, according to the manufacturer’s instructions. The content of the extracted sample was measured on a Multiskan GO microplate reader (Thermo Scientific), and each measurement was repeated three times.

### 4.4. Quantification of Hormone Contents

The six independent xylem tissues collected from high- and low-resin-yielding variants in August were used for the hormone quantification. The extraction of hormones was performed as previously described [16]. The contents of jasmonates (JAs), gibberellins (GAs), auxin, cytokinins (CTKs), 1-aminocyclopropanecarboxylic acid (ACC), salicylic acid (SA), and abscisic acid (ABA) were determined using high-performance liquid chromatography-tandem mass spectrometry (HPLC-MS/MS) on the AB SciexQTRAP 6500 LC-MS/MS platform (MetWare, Wuhan, China). The LC analytical conditions were as follows: column, Waters ACQUITY UPLC HSS T3 C18 (100 mm × 2.1 mm i.d.,1.8 µm); solvent system, water with 0.04% acetic acid (A), acetonitrile with 0.04% acetic acid (B); gradient program, started at 5% B (0–1 min), increased to 95% B (1–8 min), 95% B (8–9 min), finally ramped back to 5% B (9.1–12 min); flow rate, 0.35 mL/min; temperature, 40 °C; injection volume: 2 μL. MS/MS conditions: ion source, ESI+/−; source temperature, 550 °C; positive ion spray voltage (IS), 5500 V; negative ion spray voltage (IS), −4500 V; curtain gas (CUR) was set at 35 psi, respectively. Phytohormones were analyzed using predefined multiple reaction monitoring (MRM). Data acquisition was performed with Analyst 1.6.3 software (Sciex). Multiquant 3.0.3 software (Sciex) was used to quantify all metabolites.

### 4.5. Protein Extraction and Trypsin Digestion

In total, 12 independent xylem tissues (high- and low-yielders with three biological replicates at two stages) were used for proteomic analysis. The sample (400 mg fresh weight) was ground into powder in liquid nitrogen and transferred to 5 mL centrifuge tube. Subsequently, the sample was sonicated three times on ice with a high-intensity ultrasonic processor (Scientz, Ningbo, China) in four volumes of lysis buffer (10 mM dithiothreitol, 1% Protease Inhibitor Cocktail, and 2 mM EDTA). An equal volume of Tris-saturated phenol (pH 8.0) was added, and the mixture was then vortexed for 5 min. After centrifugation at 5000× *g* for 10 min at 4 °C, the upper phenol phase was transferred to a new centrifuge tube. The protein was precipitated by adding five volumes of 0.1 M ammonium sulfate-saturated methanol and incubated at −20 °C overnight. After centrifugation at 5000× *g* for 10 min at 4 °C, the supernatant was discarded. The remaining precipitate was washed with ice-cold methanol once, followed by ice-cold acetone three times. The protein was redissolved in 8 M urea, and the protein concentration was determined with a BCA kit according to the manufacturer’s instructions. For digestion, the protein solution was reduced with 5 mM dithiothreitol for 30 min at 56 °C and alkylated with 11 mM iodoacetamide for 15 min at room temperature in darkness. The protein sample was further diluted by adding 100 mM TEAB to urea concentration less than 2 M. Finally, trypsin was added at 1:50 trypsin-to-protein mass ratio for the first digestion overnight and 1:100 trypsin-to-protein mass ratio for a second 4 h-digestion.

### 4.6. TMT Labeling and LC-MS/MS Analysis

After trypsin digestion, peptides were desalted using Strata X C18 SPE column (Phenomenex, Torrance, CA, USA) and vacuum-dried. The peptides were reconstituted in 0.5 M TEAB, mixed with acetonitrile dissolved TMT reagent (Thermo Scientific), and then peptide mixtures were incubated for 2 h at room temperature, followed by centrifugation, desalting, and vacuum drying. The tryptic peptides were fractionated into fractions by high pH reverse-phase HPLC using Agilent 300Extend C18 column (Agilent, Santa Clara, CA, USA). Then, the peptides were dissolved in 0.1% formic acid (solvent A) and analyzed using a Q Exactive HF-X mass spectrometer coupled with an EASY-nLC 1000 UPLC system (Thermo Scientific). The mass spectrometer (MS) was operated in the data-dependent mode at an electrospray voltage of 2.0 kV. The scan range of the full scan and resolution was 350–1800 *m*/*z* and 70,000, respectively. In the MS survey scan, dynamic exclusion was 30.0 s. Automatic gain control (AGC) was set at 5 × 10^4^, fixed first mass was set as 100 *m*/*z*, and the top 20 intense ions were detected to use for MS/MS.

### 4.7. Protein Identification and Quantitation

The resulting MS/MS data was processed using MaxQuant search engine v.1.5.2.8 [65] to search against the masson pine database constructed by Iso-Seq [26]. The precursor mass tolerance was set as 20 ppm in the first search and 5 ppm in the main search, and the fragment mass tolerance was set as 0.02 Da. The variable modifications were oxidation (M), the fixed modification was carbamidomethyl (C), and up to two missed cleavages were allowed. The identified proteins contained at least one unique peptide with a false discovery rate (FDR) < 1% and the minimum score for peptides was set to >40. The quantitative level of the peptide was determined based on the intensity ratio of its ion signal in the secondary spectrum. The *t*-test analysis was used to define the proteins with significant quantitative differences between the sample pairs. Proteins with only fold change (ratio) > 1.2 and *p*-value < 0.05 were defined as differentially expressed proteins (DEPs).

### 4.8. Bioinformatics Analysis

Clusters of Orthologous Groups of proteins (COG) (https://www.ncbi.nlm.nih.gov/research/cog-project/) (accessed on 18 January 2022) and Kyoto Encyclopedia of Genes and Genomes (KEGG) (https://www.genome.jp/kegg/) (accessed on 20 January 2022) databases were used for protein family and pathway analysis [66,67]. Gene Ontology (GO) and InterPro (IPR) functions were annotated using InterProScan 5 (http://www.ebi.ac.uk/interpro/) (accessed on 4 March 2022) [68]. In addition, the WoLF PSORT tool (https://www.genscript.com/wolf-psort.html) (accessed on 17 May 2022) was used for subcellular localization prediction [69]. The functional enrichment of DEPs was performed using GO (http://www.geneontology.org/) (accessed on 14 November 2022) and KEGG databases to identify enriched functional categories and pathways [70]. A two-tailed Fisher’s exact test was employed to test the enrichment of DEPs against all identified proteins. Each GO term and KEGG pathway with a corrected *p*-value < 0.05 was considered significant. The expression trends of DEPs were analyzed based on fuzzy c-means algorithm using the online Mfuzz clustering analysis tool from PTM Cloud Platform (http://www.ptmbiolab.com) (accessed on 7 December 2022). Mufzz analysis parameters: The clustering number k is set to 8, and the clustering fuzzy degree m is set to 2.

### 4.9. Gene Expression Analysis

The quantitative real-time PCR (qRT-PCR) assay was used for the relative gene expression analysis. Total RNA was extracted using an EASYspin Plus plant RNA kit (Aidlab Biotechnologies Co., Ltd., Beijing, China), and reverse transcription was performed using a PrimeScript RT reagent Kit with gDNA Eraser (TaKaRa, Beijing, China). Primers were designed using Primer Premier 5.0 software (PREMIER Biosoft, San Francisco, CA, USA). The resulting cDNA mixture was used for subsequent analysis. qRT-PCR analysis was conducted using a CFX96 Real-Time PCR System (Bio-Rad, Hercules, CA, USA). PCR reaction system contained 1 µL of cDNA template, 5 µL of PowerUp™ SYBR™ Green Master Mix (Thermo Scientific), 0.3 µL of forward primer (10 µM), 0.3 µL of reverse primer (10 µM), and 3.4 µL of ddH_2_O. The *SKI* gene was used as the internal control [26], and the primers used in qRT-PCR are listed in Supplementary Table S8. The relative abundance of gene expression was calculated using the comparative C_T_ method [71]. Three biological replicates and three technical replicates were performed for each sample.

### 4.10. Parallel Reaction Monitoring Analysis

The six independent samples of high and low resin-yielding variants in August were used for the parallel reaction monitoring (PRM) analysis. PRM was performed in a Q Exactive HF-X mass spectrometer coupled online to the UPLC (Thermo Scientific). The mass spectrometer (MS) was operated in the data-independent procedure at an electrospray voltage of 2.1 kV. The *m*/*z* scan range was 355 to 1182 for a full scan; intact peptides were detected using Orbitrap at a resolution of 70,000, and the fragments were detected using Orbitrap at a resolution of 17,500. Automatic gain control (AGC) was set at 3 × 10^6^ for full MS and 1 × 10^5^ for MS/MS. The maximum IT was set at 50 ms for full MS and 165 ms for MS/MS. The isolation window for MS/MS was set at 1.6 *m*/*z*. Ion activation/dissociation was performed at normalized collision energy of 27 in a higher energy dissociation (HCD) collision cell. The raw data were analyzed using Skyline 20.2 (MacCoss Lab, University of Washington). Peptide settings: enzyme was set as Trypsin [KR/P], max missed cleavage set as 0. The peptide length was set as 7–25, variable modification was set as carbamidomethyl on Cys and oxidation on Met, and max variable modifications were set as 3. Transition settings: precursor charges were set as 2, 3, ion charges were set as 1, and ion types were set as b, y. The product ions were set from ion 3 to the last ion, and the ion match tolerance was set as 0.02 Da.

### 4.11. Statistical Analysis

Statistical analyses were performed with SPSS 22.0 software (SPSS Inc., Chicago, IL, USA). The single-factor analysis of variance (ANOVA) was employed to determine the differences between sample pairs, and *p* < 0.05 and *p* < 0.01 were considered significant and extremely significant, respectively. All data were represented by at least three repeated means.

## 5. Conclusions

**Figure 11:**
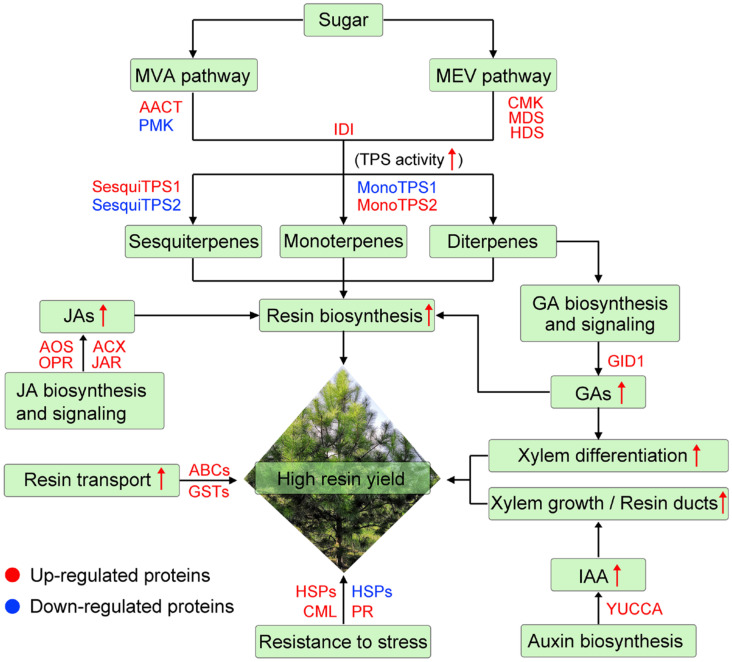
The regulatory diagram of resin yield in masson pine. The red arrow indicates that the content/activity of the substance is increased or the metabolic pathway is enhanced. HSPs contain both up- and down-regulated proteins.

In this study, the mechanisms regulating resin yield were investigated at the physiological and molecular levels. Resin yield could be regulated by carbohydrate metabolism, terpenoid biosynthesis, resistance to stress, and phytohormone biosynthesis and signaling. The carbohydrate metabolism and terpenoid biosynthesis may positively regulate resin yield by promoting resin biosynthesis. Stress-related proteins may influence resin yield by regulating responses to stress. JA and GA biosynthesis and signaling may regulate resin yield by modulating terpenoid biosynthesis. Additionally, GA may regulate resin yield by promoting xylem differentiation. Auxin may regulate resin yield by participating in xylem growth and resin duct development. By further analyzing the expression levels of DEPs involved in these regulatory pathways, a set of proteins associated with resin yield were identified, including terpenoid backbone biosynthesis-related proteins (e.g., HDS, IDI, and FPPS), TPSs, ABCs, GSTs, HSPs, and phytohormone biosynthesis and signaling-related proteins (e.g., OPR, JAR, and GID1). Relative expression analysis of resin yield-related genes suggested that these gene expressions were also associated with resin yield. Overall, our results provide new insights into the molecular mechanisms regulating resin yield, shedding light on the genetic improvement in masson pine and the utilization of valuable genetic resources (Figure 11).

## Data Availability

All data supporting the findings of this study are included within the article (and its Supplementary Materials files).

## Supplementary Materials

The supporting information can be downloaded at: https://www.mdpi.com/article/10.3390/ijms241813813/s1.
