# Supplementary material for: Physiological, Proteomic, and Resin Yield-Related Genes Expression Analysis Provides Insights into the Mechanisms Regulating Resin Yield in Masson Pine
Source: Int J Mol Sci. 2023 Sep 7;24(18):13813. doi: 10.3390/ijms241813813 (PMC10531451; doi:10.3390/ijms241813813)
Supplement: Supplementary file 1 [file ijms-24-13813-s001.zip › Figure S1.pdf]

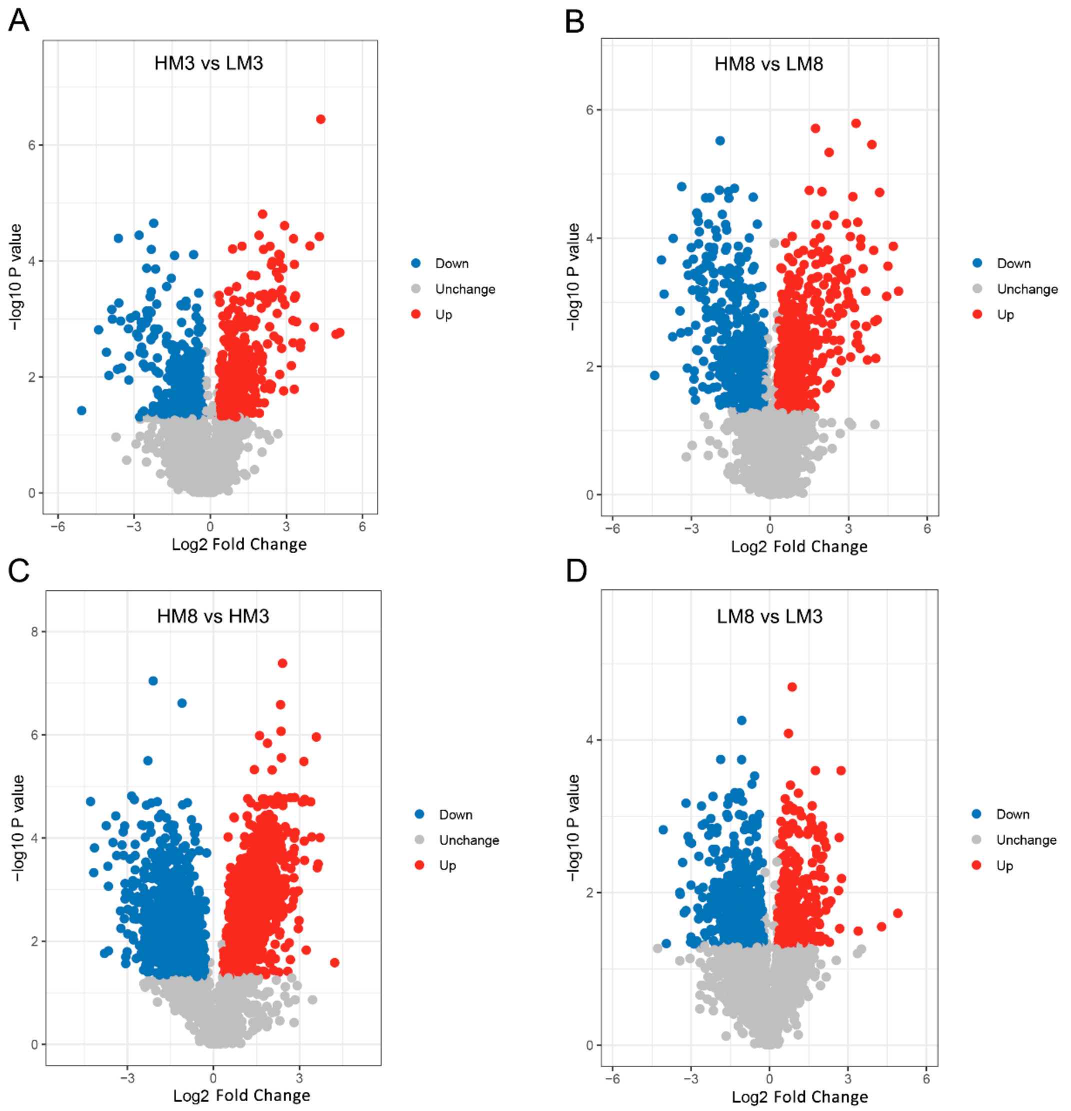

Figure S1. Volcano plots in HM3 vs. LM3 (A), HM8 vs. LM8 (B), HM8 vs. HM3 (C), and LM8 vs. LM3 (D). Red spots represent significant up-regulated proteins, blue spots represent significant down-regulated proteins, and gray spots represent nonsignificant regulated proteins.
