# Supplementary material for: Physiological, Proteomic, and Resin Yield-Related Genes Expression Analysis Provides Insights into the Mechanisms Regulating Resin Yield in Masson Pine
Source: Int J Mol Sci. 2023 Sep 7;24(18):13813. doi: 10.3390/ijms241813813 (PMC10531451; doi:10.3390/ijms241813813)
Supplement: Supplementary file 1 [file ijms-24-13813-s001.zip › Figure S2.pdf]

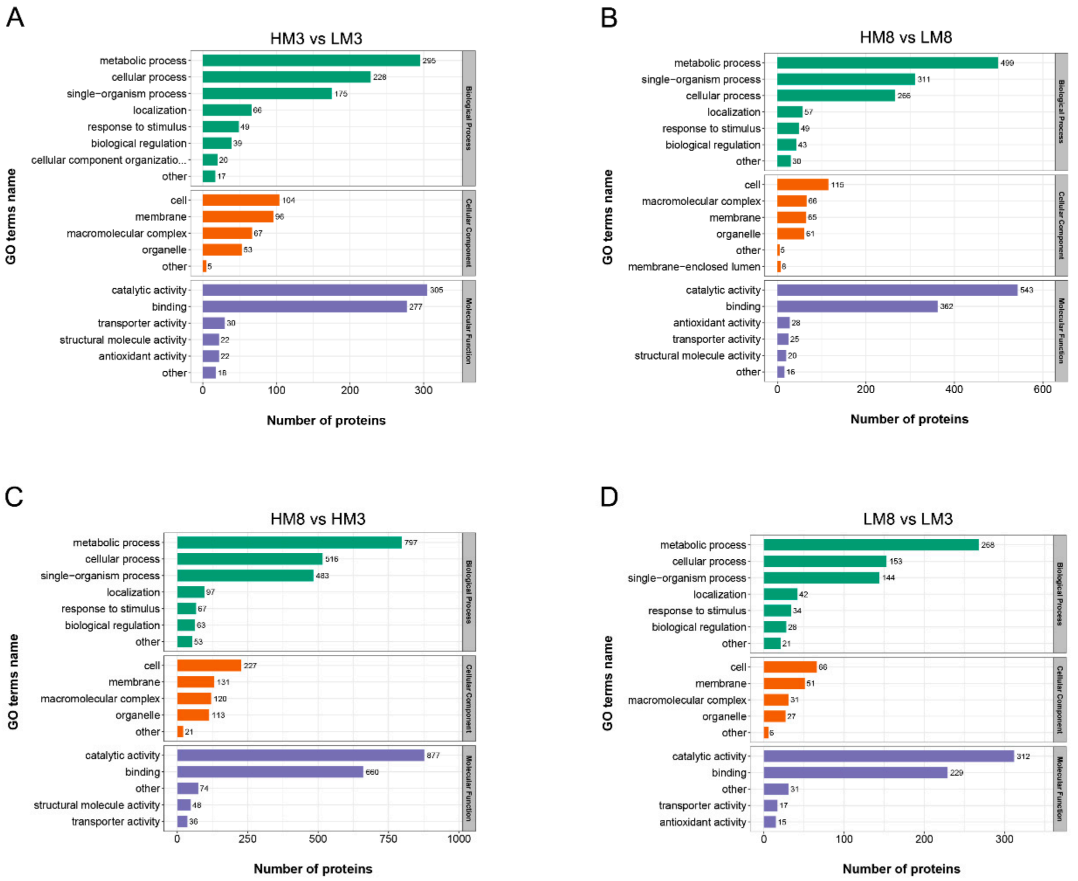

Figure S2. GO functional classification of DEPs in different comparison groups. (A) DEPs enriched in HM3 vs. LM3. (B) DEPs enriched in HM8 vs. LM8. (C) DEPs enriched in HM8 vs. HM3. (D) DEPs enriched in LM8 vs. LM3.
