# Supplementary figures and images for: Physiological, Proteomic, and Resin Yield-Related Genes Expression Analysis Provides Insights into the Mechanisms Regulating Resin Yield in Masson Pine
Source: Int J Mol Sci. 2023 Sep 7;24(18):13813. doi: 10.3390/ijms241813813 (PMC10531451; doi:10.3390/ijms241813813)

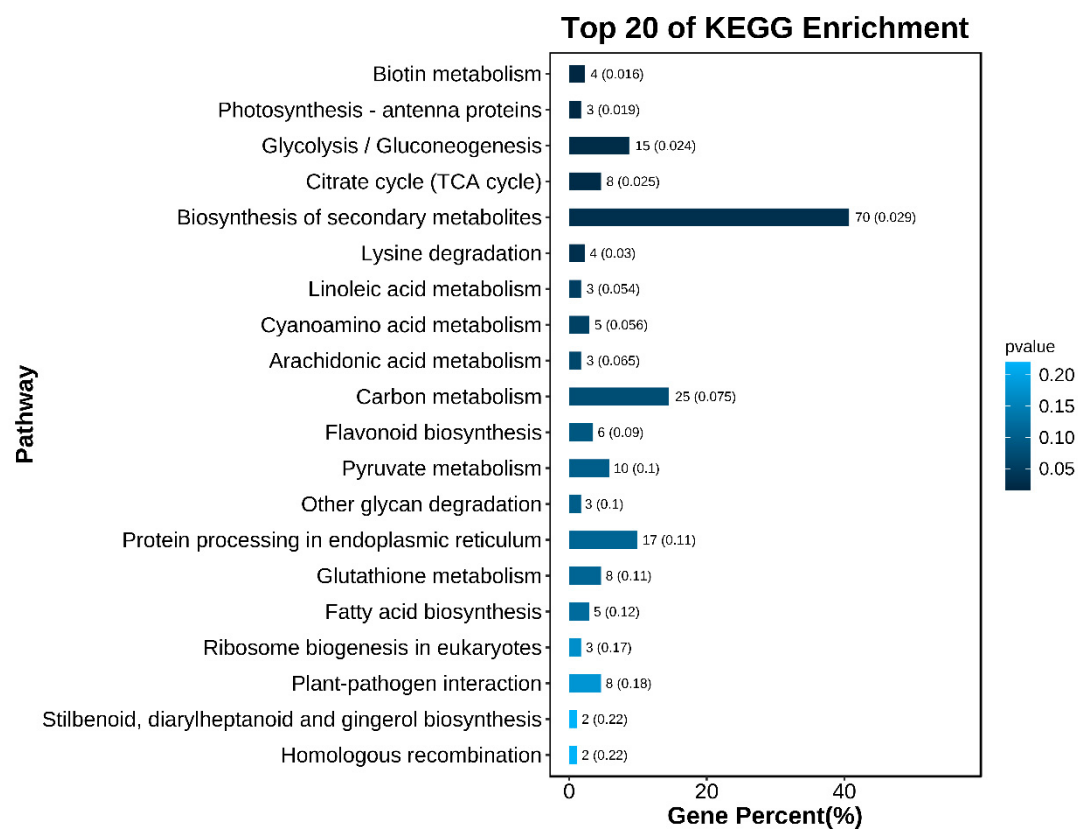

**Figure S3.** KEGG enrichment pathways of DEPs in clusters 4, 7, 1, and 5.

Supplement: Supplementary file 1 [file ijms-24-13813-s001.zip › Figure S3.pdf]
